# Analysis of pulmonary nodules in patients with high-grade soft tissue sarcomas

**DOI:** 10.1371/journal.pone.0172148

**Published:** 2017-02-09

**Authors:** Tomoki Nakamura, Akihiko Matsumine, Miki Matsusaka, Keitaro Mizumoto, Mayuko Mori, Tomoya Yoshizaki, Takao Matsubara, Kunihiro Asanuma, Akihiro Sudo

**Affiliations:** 1 Department of Orthopaedic Surgery, Mie University Graduate School of Medicine, Tsu-city, Mie, Japan; 2 Medical student, Mie University School of Medicine, Tsu-city, Mie, Japan; Ospedale Pediatrico Bambino Gesu, ITALY

## Abstract

Nowadays, small pulmonary nodules are easily detectable in patients with soft tissue sarcomas (STSs) because of highly improved computed tomography (CT) technologies. The purpose of this study was to determine the frequency and significance of the pulmonary nodules detected by CT in high-grade STS patients. 124 patients with high-grade STS were retrospectively reviewed. There were 72 males (57%) and 52 females (43%). Patients’ average age was 61 years (median (quartiles) 66 years (48–75), range 8–94 years). Pulmonary nodules were detected in 49 (39.5%) of 124 patients by CT scanning at first presentation. Of 49 patients with nodules at first presentation, 34 (69.4%) had benign lesions, and 13 (26.5%) had metastatic nodules. One patient (2%) had primary lung cancer and the remaining one with one nodule could not be definitively diagnosed due to a short follow-up time. 30 patients (24.1%) of 124 patients developed pulmonary nodules during their clinical progression. Seven (23.3%) had benign lesions, whereas 21 (70%) had metastatic lesions. Primary lung cancer was detected in two patients (6.7%). The size and timing of detection of a pulmonary nodule significantly affected the final clinical diagnosisby multivariate analysis. We conclude that pulmonary nodules can be detected highly frequently in patients with high-grade STSs because of improved CT technologies. Careful follow-up is needed if nodules are detected after initial treatment or during the clinical course of the disease.

## Introduction

Nowadays, small pulmonary nodules are easily detectable in patients with soft-tissue sarcomas (STSs) because of highly improved computed tomography (CT) technologies [1–5]. For example, by using thin-section CT (i.e., 2 mm slice thickness), Hanamiya et al. reported the rate of detection of the non-calcified pulmonary nodules to be 75% in patients with extra-pulmonary malignant tumors [5]. ^18^F-fluorodeoxyglucose positron emission tomography (FDG-PET) also has been increasingly used for screening patients. However, FDG-PET sensitivity can be adversely affected by the size of a metastatic pulmonary nodule. Reinhart et al. reported that only 39.7% of 439 metastatic pulmonary lesions ranging from 3 to 60 mm in diameter could be positively detected by FDG-PET [6]. And in sarcoma cases, Fortes et al. reported that only 44% of pulmonary nodules could be detected positively with FDG-PET [7]. Consequently, CT has become the standard diagnostic tool for detecting small pulmonary lesions.

We previously reported that 70 (29.4%) out of 206 patients with bone or soft tissue sarcomas developed pulmonary nodules that could be detected by CT. We used a CT slice thickness of ≥ 7 mm in 56% of the 70 patients (unpublished data) [3]. Recently, it has become common to use a slice thickness of ≤ 5 mm for chest CT screening. Therefore, we hypothesize that the CT detection rate of pulmonary nodules may be higher than that previously reported.

Patients with high-grade tumors are at significant risk of relapse, and as many as 50% of them consequently die [8]. Furthermore, of all the sarcoma patients, 62–83% present with lung metastases, and 50–70% show isolated lung metastases [9]. Therefore, determining whether a pulmonary nodule is malignant or benign is critical to subsequently indicating additional treatments, including surgical resection and/or chemotherapy.

The purpose of this study was to determine the frequency and significance of the pulmonary nodules detected by CT in high-grade STS patients.

## Patients and methods

**Fig 1:**
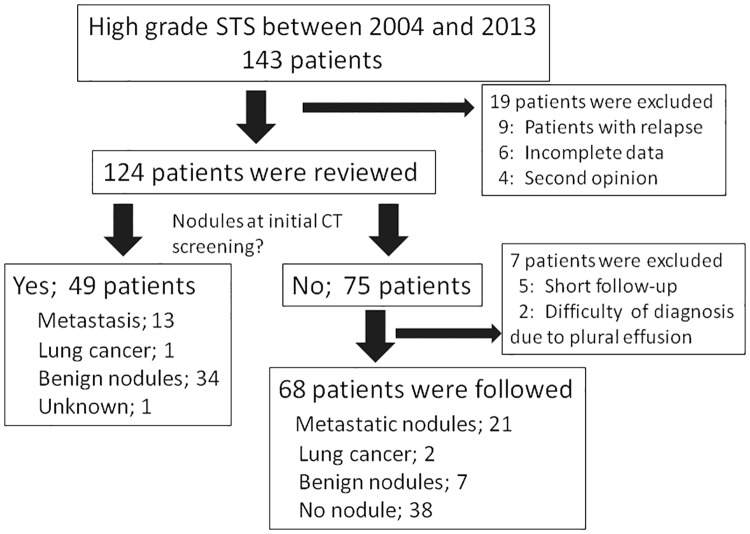
Study profile.

One hundred and forty-three patients with a recent diagnosis of high-grade STS were referred to our hospital. The medical records of those with histologically confirmed high-grade primary STSs were reviewed from January 2004 to December 2013. The high grade was defined as grade 2 or 3 according to the grading system by the French Federation of Cancer Centers Sarcoma Group (FNCLCC). Nine patients with local recurrence or metastasis were excluded. Six other patients were excluded due to incomplete records. Four patients who were referred for second opinion were also excluded. Finally, 124 patients were retrospectively reviewed (Fig 1). Of 124 patients, 37 (29.8%) were studied in our previous report [3].

To determine the presence of the pulmonary nodules at first presentation, patients’ clinical records and their corresponding chest CT scans were reviewed first. If any pulmonary nodule was found, their number, size, and distribution were recorded.

If pulmonary nodules were absent at first presentation, patients’ chest CT scans were reviewed after the initial treatment to determine presence or absence of nodules.

Chest CT images (obtained with X-Vigor or Aquilion, Toshiba, Tokyo, Japan; VCT or CT750HD, GE, Connecticut, USA) were used to detect potential pulmonary nodules. In addition to the initial radiologists’ assessments, patients’ chest CT results were reviewed by one orthopaedic oncologist and two medical students who were consisted into four orthopedic oncologists (T. N., T. M., K. A., and A. M.) and four medical students (M. M., K. M., M. M., and T. Y.) to determine the number, size, and distribution of nodules. This re-examination was necessary because small nodules were not routinely reported by the radiologists.

Pulmonary nodules were diagnosed as either a “metastatic pulmonary lesion” or a “benign pulmonary lesion”.

“Metastatic pulmonary lesions” were defined based on the presence of at least one of the following criteria: 1) histological examinations confirmed a metastatic lesion in specimens obtained by either CT-guided needle biopsy or surgical resection; 2) lesions showed an obvious progression in number and/or size; 3) extra-pulmonary metastatic lesions appeared during the follow-up period [3,10,11].

“Benign pulmonary lesions” were defined based on the presence of at least one of the following criteria: 1) histological examinations confirmed a benign lesion in specimens obtained by CT-guided needle biopsy or surgical resection; 2) lesions showed no progression at least for one year [3,10,11].

The statistical associations between the clinicopathological factors were evaluated using the Mann–Whitney U-test for quantitative data, and the Χ^2^ test for qualitative data. Univariate and Multivariate analyses were performed using a logistic regression model. In multivariate analysis, all the parameters with p < 0.3 in univariate analysis were included. P < 0.05 was considered to be significant in all statistical analyses. The statistical software package Stat View, version 5.0 (SAS Institute Inc Cary, NC, USA), was used. This study was approved by the institutional review board at Mie University Hospital (ID2867). Writen informed consent for participants was not obtained because institutional review board waived this requirement due to the retrospective nature of this study.

## Results

### Detection rate of pulmonary nodules

There were 72 males (57%) and 52 females (43%). Patients’ average age was 61 years (median (quartiles) 66 years (48–75), range 8–94 years).

Pulmonary nodules were detected in 49 of 124 patients (39.5%, 49/124)) by CT scanning at first presentation (Fig 1). The CT slice thickness used at the initial screening was: < 5 mm (n = 29), = 5 mm (n = 67), and > 5 mm (n = 28). Of 75 patients (60.5%, 75/124) who did not show any nodule at the initial screening, seven patients were excluded from further study because five could not be followed up, and two developed acute hydrothorax (probably metastasis) on final CT scans. Therefore, 68 patients were followed; 30 (44.1%) developed pulmonary nodules during their clinical progression. Pulmonary nodules were observed in 79 patients (63.7%, 79/124) at the last review date (December, 2014). Age (p = 0.64) and gender (p = 0.42) were not associated with the detection of pulmonary nodules.

### Diagnosis of pulmonary nodules in 49 patients at initial screening

Of 49 patients with nodules at first presentation, 34 had benign lesions, and 13 had metastatic nodules. Primary lung cancer was detected in one patient. One nodule could not be definitively diagnosed due to a short follow-up time.

Metastatic lesions were confirmed through histology by using specimens obtained with CT-guided needle biopsy or surgical resection in four of 13 patients. Nine patients’ lesions had progressed in number and/or size. One patient had a benign lesion by using specimens obtained with surgical resection. Thirty-three patients did not show progression at least during one year of follow-up.

Of 34 patients with benign pulmonary nodules, 10 developed new nodules. One patient was diagnosed with a benign lesion, but nine had metastases.

### Clinical course of the patients who did not have pulmonary nodules at initial presentation

Of 68 patients who did not have pulmonary nodules at initial screening, 30 (44.1%) developed pulmonary nodules: seven had benign lesions, whereas 21 had metastatic lesions. Primary lung cancer was detected in two patients. Seven patients had histologically confirmed metastatic lesions. Fourteen patients showed progression in nodule number and/or size. One patient had a histologically confirmed benign lesion. Six patients did not show any progression during at least one year of follow-up. No nodule could be detected on CT scanning in the remaining 38 patients.

### Analysis of patients with pulmonary nodules

Pulmonary nodules were detected in 79 patients. One patient was excluded from this analysis due to difficulty of final diagnosis. Three patients with primary lung cancers were also excluded from further analysis. CT slice thickness used to detect pulmonary nodules in the 75 patients was: < 5 mm (n = 32), = 5 mm (n = 32), > 5 mm (n = 11).

Relationships between the final clinical diagnosis of pulmonary nodules and clinical characteristics, including patients’ age, sex, number, the timing at nodule, distribution and nodule size are summarized in Table 1. The size, number, and timing of detection of a pulmonary nodule were significantly associated with the status of the nodule being malignant or benign by univariate analysis. The median size of the largest pulmonary nodule was 6.1 (quartiles, 5–9.4) and 3.5 (2.5–4) mm in malignant nodules (including metastasis and primary lung cancer) and benign nodules (P < 0.0001), respectively. Of 20 patients with pulmonary nodules ≤ 3 mm, four (20%) had malignant nodules. Of 49 patients who had nodules ≤ 5 mm, 16 (32.7%) had malignant nodules. In contrast, 92.3% (24 of 26 patients) who had nodules > 5 mm had malignant nodules. The median number of malignant and benign nodules were 2 (quartiles, 1–5) and 1(1–2) (p = 0.002), respectively. Pulmonary nodules which were detected during the patients’ clinical course of disease were likely to be malignant as opposed to those detected at first presentation (p < 0.0001).

**Table 1:** The relationship between the final diagnosis and clinical features.

Variables		Diagnosis at initial pulmonary nodules		p value
		Metastasis (n = 34)	Benign (n = 41)	
Age (years)	Median (quartiles)	68 (45–82)	64 (47–73)	0.23
Gender	Male	18	27	0.26
	Female	16	14	
Size(mm)	Median (quartiles)	6.1 (5–9.4)	3.5 (2.5–4)	<0.0001
Number	Median (quartiles)	2 (1–5)	1 (1–2)	0.0008
Timing at nodule	Initial screening	13	34	<0.0001
	Clinical course	21	7	

Using logistic regression model, tumor size (p = 0.001), number (p = 0.01), distribution (p = 0.0002) and timing of detection (p = 0.0002) were significant factors for diagnosis of metastasis in univariate analysis (Table 2).

**Table 2:** Logistic univariate analysis for diagnosis of pulmonary nodules.

Variables		OR	95% CI	p value
Size	(mm)	2.309	1.514–3.52	0.0001
Number		1.39	1.072–1.803	0.01
Timing of nodules	initial screening	0.127	0.044–0.371	0.0002
Distribution of nodules	unilateral	0.11	0.035–0.358	0.0002
Age	(years)	1.009	0.988–1.031	0.39
Gender	Male	0.583	0.229–1.483	0.26

OR; Odds ratio, 95% CI; 95% Confidential interval

In multivariate analyses, timing of detection and size of the pulmonary nodules remained significant factors (p = 0.008 and p = 0.001, respectively) (Table 3).

**Table 3:** Logistic multivariate analysis for diagnosis of pulmonary nodules*.

Variables		OR	95% CI	p value
Size	(mm)	2.289	1.389–3.773	0.001
Number		1.113	0.704–1.389	0.65
Timing of nodules	initial screening	0.121	0.025–0.580	0.008
Distribution of nodules	unilaretal	0.211	0.023–1.951	0.17
Gender	Male	0.43	0.084–2.204	0.31

OR; Odds ratio, 95% CI; 95% Confidential interval

*Predictive factors include al parameters with p value < 0.3 in univariate analysis.

### Analysis of patients without pulmonary nodules

No nodules were detected in 38 patients by CT scanning. The mean follow-up time was 40.1 months (median; 31.4 months, quartiles; 18.7–55.4). Mean number of lung CT scans were 7 (median; 8, quartiles; 5.3–9). The thickness of the CT slice during patients’ follow-up period was: < 5 mm (n = 33), = 5 mm (n = 4), > 5 mm (n = 1).

## Discussion

Lungs are the most common sites of STS metastases. The detection rate of small pulmonary nodules has been increasing due to improvements in various diagnostic modalities [1–5]. The detection rate of nodules using CT with 10mm slice thickness was 23% in the Early Lung Cancer Action Project [12]. Swensen et al. reported in 2005 the detection rate of one or more nodules among smokers aged 50 and older to be 74% in low-dose screening CT with 3.75 mm thickness [13]. Furthermore, recent study showed 75% of the patients with extra-pulmonary malignant tumors were detected using CT with 2 mm thickness [5]. Of these, CT has been the standard diagnostic test for detecting small pulmonary lesions [1–5,12–13]. CT is limited in allowing differential diagnosis between the malignant or metastatic lesions and benign nodules. In this study, pulmonary nodules were identified in 39.5% (49/124) of patients with high-grade STSs at initial presentation, and 63.7% (79/124) at follow-up periods. These numbers indicate a high rate of successful detection of nodules in high-grade STS patients. This may partially be because we used a CT slice thickness of ≤ 5 mm for detecting nodules in 83% of 78 patients who had pulmonary nodules. In fact, the detected pulmonary nodules were ≤3 mm in size in 20 of 78 patients, and 80% of the 20 patients were diagnosed as benign.

Pulmonary metastasis was observed in 10.5 and 34.5% of patients at initial presentation and follow-up time, respectively. These rates are similar to other reported rates [1–3,14–16]. Interestingly, 70% of 30 patients which developed lung nodules after their initial treatment were diagnosed to have metastases. Furthermore, 10 of 34 patients who had benign nodules at initial presentation developed new nodules, and 9 of 10 patients had metastatic tumors at different lung regions. Therefore, careful follow-up is needed if a nodule is detected during the clinical course of the disease after initial treatment.

Additionally, the nodule size was also a predictive factor for diagnosis. The rate of malignancy was higher in patients with nodules > 5 mm in size. Rissing et al. reported that patients with indeterminate nodules of ≥ 5 mm in size have a higher risk for developing metastatic disease than those with normal CCT, or those with nodules < 5 mm [2]. We previously reported statistically significant differences between overall survival rates of patients with nodules ≤ 5 mm in size and those with larger nodules in 206 patients with bone sarcomas or STSs [3]. In this study, our CT screening at ≤ 5 mm slice thickness detected nodules in 85.3% of patients who developed pulmonary nodules at initial screening, and 97.4% of patients who developed nodules during their follow-up. Therefore, the rate of successful detection of small (≤ 5 mm) metastatic nodules is likely increasing. However, small metastatic nodules cannot be differentially diagnosed by a single CT screening and thus should be followed up. Our findings suggest that biopsy or surgical resection should be considered if the size of a pulmonary nodule exceeds 5 mm.

Lillington et al. noted that multiple nodules may indicate a greater likelihood of developing the pulmonary metastatic disease in adult patients [17]. However, this study found that the number of nodules was not a significant factor in multivariate analysis.

One limitation of this study is its retrospective nature. Another limitation is that benign pulmonary nodules may be misdiagnosed clinically. Furthermore, although the frequency of CT examinations depends on the physician, pretreatment workup, including lung CT and follow-up CT every 3–6 months, were routinely performed at our hospital.

## Conclusions

We conclude that pulmonary nodules can be detected highly frequently in patients with high-grade STSs because of improved CT technologies. Careful follow-up is needed if nodules are detected after initial treatment or during the clinical course of the disease. Biopsy or surgical resection should be considered if the size of the pulmonary nodules exceeds 5 mm.

## Supporting information

S1 Table The profile of 75 patients who had benign and metastatic nodules. (DOC)

## References

[1] Ferguson PC, Deheshi BM, Chung P, Catton CN, O’Sullivan B, Gupta A, et al Soft tissue sarcoma presenting with metastatic disease: outcome with primary surgical resection. Cancer 2011; 117: 372–379. 10.1002/cncr.25418 20830769

[2] Rissing S, Rougraff BT, Davis K. Indeterminate pulmonary nodules in patients with sarcoma affect survival. Clin Orthop Relat Res 2007; 459: 118–121. 10.1097/BLO.0b013e31805d8606 17438474

[3] Nakamura T, Matsumine A, Niimi R, Matsubara T, Kusuzaki K, Maeda M, et al Management of small pulmonary nodulesin patients with sarcoma. Clin Exp Metastasis 2009; 26: 713–718. 10.1007/s10585-009-9270-y 19466567

[4] Billingsley KG, Burt ME, Jara E, Ginsberg RJ, Woodruff JM, Leung DH, et al Pulmonary metastases from soft tissue sarcoma: analysis of patterns of disease and postmetastasis survival. Ann Surg 1999; 229: 602–612. 10235518 10.1097/00000658-199905000-00002 PMC1420804

[5] Hanamiya M, Aoki T, Yamashita Y, Kawanami S, Korogi Y. Frequency and significance of pulmonary nodules on thin-section CT in patients with extrapulmonary malignant neoplasms. Eur J Radiol 2012; 81: 152–157. 10.1016/j.ejrad.2010.08.013 20828958

[6] Reinhardt MJ, Wiethoelter N, Matthies A, Joe AY, Strunk H, Jaeger U, et al PET recognition of pulmonary metastases on PET/CT imaging: impact of attenuation-corrected and non attenuation-corrected PET images. Eur J Nucl Med Mol Imaging 2006; 33: 134–139. 10.1007/s00259-005-1901-1 16193313

[7] Fortes DL, Allen MS, Lowe VJ, Shen KR, Wigle DA, Cassivi SD, et al The sensitivity of 18F-fluorodeoxyglucose positron emission tomography in the evaluation of metastatic pulmonary nodules. Eur J Cardiovasc Surg 2008; 34: 1223–1227. 10.1016/j.ejcts.2008.09.007 18848459

[8] Kattan MW, Leung DH, Brennan MF. Postoperative nomogram for 12-year sarcoma-specific death. J Clin Oncol 2002; 20: 791–796. 11821462 10.1200/JCO.2002.20.3.791

[9] Nakamura T, Matsumine A, Matsubara T, Asanuma K, Niimi R, Uchida A, et al Retrospective analysis of metastatic sarcoma patients. Oncol Lett 2011; 2: 315–318. 10.3892/ol.2011.238 22866083 PMC3410588

[10] Lagaru A, Chawla S, Menendez L, Conti PS. ^18^F-PDG PET and PET/CT for detection of pulmonary metastases from musculoskeletal sarcomas. Nuclear Med Commun 2006; 27: 795–802. 10.1097/01.mnm.0000237986.31597.86 16969262

[11] Bacci G, Mercuri M, Briccoli A. Osteogenic sarcoma of the extremity with detectable lung metastases at presentation. Cancer 1997; 79: 245–254. 9010097

[12] Henschke CI, McCauley DI, Yankelevitz DF, Naidich DP, McGuinness G, Miettinen OS, et al Early lung cancer action project: overall design and findings from baseline screening. Lancet 1999; 354: 99–105. 10.1016/S0140-6736(99)06093-6 10408484

[13] Swensen SJ, Jett JR, Hartman TE, Midthun DE, Mandrekar SJ, Hillman SL, et al CT screening for lung cancer: five-year prospective experience. Radiology 2005; 235: 259–265. 10.1148/radiol.2351041662 15695622

[14] Kane JM, Finley JW, Driscoll D, Kraybill WG, Gibbs JF. The treatment and outcome of patients with soft tissue sarcomas and synchronous metastases. Sarcoma 2002; 6: 69–73. 10.1080/1357714021000022168 18521331 PMC2395477

[15] Pollock RE, Karnell LH, Menck HR, Winchester DP. The National Cancer Data Base report on soft tissue sarcoma. Cancer 1996; 78: 2247–2257. 8918421

[16] Zagars GK, Ballo MT, Pisters PW, Pollock RE, Patel SR, Benjamin RS, et al Prognostic factors for patients with localized soft-tissue sarcoma treated with conservation surgery and radiation therapy: an analysis of 1225 patients. Cancer 2003; 97: 2530–2543. 10.1002/cncr.11365 12733153

[17] Lillington GA, Caskey CI. Evaluation and management of solitary and multiple pulmonary nodules. Clin Chest Med 1993; 14: 111–119. 8462244

