# Supplementary material for: Analysis of pulmonary nodules in patients with high-grade soft tissue sarcomas
Source: PLoS One. 2017 Feb 9;12(2):e0172148. doi: 10.1371/journal.pone.0172148 (PMC5300188; doi:10.1371/journal.pone.0172148)
Supplement: S1 Table — (DOC) [file pone.0172148.s001.doc]

**Supplemental Table.**

The profile of 75 patients who had benign and metastatic nodules.

| No | Age | Gender | Nodule | Size | Distribution | Diagnosis | Nodule at |
| --- | --- | --- | --- | --- | --- | --- | --- |
|  | (years) |  |  | (mm) |  | for initial nodule | first presentation |
| 1 | 88 | Female | 1 | 3.1 | Uni | Benign | Yes |
| 2 | 38 | Male | 1 | 2 | Uni | Benign | Yes |
| 3 | 56 | Male | 1 | 2 | Uni | Benign | No |
| 4 | 84 | Male | 4 | 14 | Bi | Metastasis | No |
| 5 | 71 | Male | 1 | 2 | Uni | Benign | Yes |
| 6 | 29 | Male | 8 | 2 | Bi | Benign | No |
| 7 | 13 | Male | 2 | 6.2 | Bi | Metastasis | Yes |
| 8 | 66 | Male | 1 | 3.8 | Uni | Benign | Yes |
| 9 | 19 | Female | 1 | 3.5 | Uni | Benign | Yes |
| 10 | 64 | Male | 1 | 4.4 | Uni | Benign | Yes |
| 11 | 33 | Male | 1 | 5.2 | Uni | Metastasis | No |
| 12 | 82 | Male | 1 | 3.5 | Uni | Benign | Yes |
| 13 | 60 | Female | 18 | 11 | Bi | Metastasis | Yes |
| 14 | 69 | Female | 1 | 5 | Uni | Metastasis | Yes |
| 15 | 44 | Male | 2 | 5 | Bi | Metastasis | No |
| 16 | 45 | Male | 1 | 2.3 | Uni | Benign | Yes |
| 17 | 38 | Female | 1 | 7.8 | Uni | Metastasis | Yes |
| 18 | 86 | Female | 2 | 10 | Bi | Metastasis | No |
| 19 | 46 | Male | 1 | 6 | Uni | Metastasis | No |
| 20 | 57 | Male | 1 | 2.2 | Uni | Benign | Yes |
| 21 | 58 | Male | 1 | 3 | Uni | Benign | No |
| 22 | 73 | Female | 2 | 8 | Bi | Metastasis | No |
| 23 | 47 | Male | 1 | 6 | Uni | Benign | Yes |
| 24 | 34 | Male | 1 | 6 | Bi | Metastasis | No |
| 25 | 90 | Female | 1 | 14 | Uni | Metastasis | No |
| 26 | 21 | Female | 3 | 4 | Bi | Benign | Yes |
| 27 | 76 | Male | 3 | 4 | Uni | Benign | Yes |
| 28 | 76 | Female | 2 | 4.6 | Uni | Benign | Yes |
| 29 | 66 | Male | 1 | 3.5 | Uni | Benign | Yes |
| 30 | 76 | Male | 4 | 5.5 | Bi | Metastasis | Yes |
| 31 | 48 | Male | 7 | 3.5 | Bi | Benign | No |
| 32 | 12 | Female | 1 | 3.3 | Uni | Benign | Yes |
| 33 | 77 | Female | 18 | 14 | Bi | Metastasis | Yes |
| 34 | 72 | Male | 2 | 4.6 | Uni | Benign | Yes |
| 35 | 82 | Male | 1 | 6 | Uni | Metastasis | No |
| 36 | 74 | Female | 20 | 30 | Bi | Metastasis | No |
| 37 | 66 | Male | 2 | 4 | Uni | Benign | Yes |
| 38 | 69 | Male | 2 | 4.8 | Uni | Benign | No |
| 39 | 78 | Female | 2 | 3.8 | Uni | Benign | Yes |
| 40 | 74 | Male | 2 | 3.4 | Uni | Benign | Yes |
| 41 | 60 | Male | 10 | 9 | Bi | Metastasis | Yes |
| 42 | 75 | Male | 1 | 4.7 | Uni | Benign | Yes |
| 43 | 49 | Female | 5 | 3.5 | Bi | Metastasis | No |
| 44 | 75 | Male | 9 | 5.5 | Bi | Metastasis | No |
| 45 | 67 | Male | 2 | 3.5 | Uni | Metastasis | No |
| 46 | 35 | Male | 1 | 2.6 | Uni | Metastasis | Yes |
| 47 | 80 | Female | 1 | 20 | Uni | Metastasis | Yes |
| 48 | 16 | Male | 1 | 3 | Uni | Benign | Yes |
| 49 | 90 | Female | 11 | 4.5 | Bi | Metastasis | No |
| 50 | 21 | Female | 1 | 2.5 | Uni | Benign | Yes |
| 5 | 56 | Female | 1 | 3 | Uni | Benign | No |
| 52 | 89 | Male | 2 | 4 | Bi | Benign | Yes |
| 53 | 67 | Male | 1 | 9 | Uni | Metastasis | No |
| 54 | 66 | Female | 1 | 2 | Uni | Benign | Yes |
| 55 | 64 | Female | 2 | 9.5 | Uni | Metastasis | No |
| 56 | 37 | Male | 2 | 7.5 | Bi | Metastasis | Yes |
| 57 | 61 | Male | 4 | 2 | Bi | Metastasis | No |
| 58 | 18 | Female | 1 | 1.7 | Uni | Metastasis | No |
| 59 | 65 | Female | 2 | 4 | Uni | Benign | Yes |
| 60 | 63 | Male | 1 | 2.6 | Uni | Benign | Yes |
| 61 | 80 | Male | 1 | 2.5 | Uni | Benign | Yes |
| 62 | 85 | Male | 5 | 9 | Bi | Metastasis | Yes |
| 63 | 92 | Male | 2 | 11 | Uni | Metastasis | No |
| 64 | 86 | Female | 1 | 5.7 | Uni | Metastasis | No |
| 65 | 85 | Male | 1 | 4.5 | Uni | Benign | No |
| 66 | 71 | Female | 11 | 8 | Bi | Metastasis | No |
| 67 | 69 | Male | 2 | 2 | Bi | Benign | Yes |
| 68 | 49 | Female | 1 | 3.5 | Uni | Benign | Yes |
| 69 | 36 | Female | 1 | 2.5 | Uni | Benign | Yes |
| 70 | 89 | Female | 2 | 4 | Uni | Metastasis | Yes |
| 71 | 8 | Male | 10 | 3 | Bi | Metastasis | Yes |
| 72 | 51 | Male | 1 | 3.1 | Uni | Benign | Yes |
| 73 | 29 | Female | 1 | 2 | Uni | Benign | Yes |
| 74 | 73 | Male | 1 | 3.6 | Uni | Benign | Yes |
| 75 | 64 | Female | 1 | 6.5 | Uni | Benign | Yes |
| Uni; Unilateral, Bi; Bilateral | | | |  |  |  |  |
